# 1-(2,4-Dichloro­benz­yl)pyridinium bis­(2-sulfanyl­idene-1,3-dithiole-4,5-dithiol­ato-κ^2^ *S*,*S*′)nickelate(III)

**DOI:** 10.1107/S1600536811045004

**Published:** 2011-11-02

**Authors:** Guang-Xiang Liu

**Affiliations:** a Department of Chemistry, Nanjing Xiaozhuang University, Nanjing 211171, People’s Republic of China

## Abstract

In the title compound, (C_12_H_10_Cl_2_N)[Ni(C_3_S_5_)_2_], the Ni^III^ atom is chelated by two bidentate 2-sulfanyl­idene-1,3-dithiole-4,5-dithiol­ate (dmit) dianions and shows a distorted square-planar geometry. The two dmit ligands are twisted with respect to each other by 3.21 (2)°. In the cation, the two aromatic groups linked by the methyl­ene bridging group form a dihedral angle of 68.09 (2)°. S⋯S [3.6212 (11) and 3.5573 (9) Å] and Ni⋯S [3.566 (2)Å] inter­actions influence the arrangement of the anions in the crystal.

## Related literature

For potential applications of bis­(dithiol­ate)-metal complexes, see: Cassoux (1999 ▶). For the oxidation of Ni^II^ compounds, see: Cassoux *et al.* (1991 ▶). For the synthesis, see: Wang *et al.* (1998 ▶).
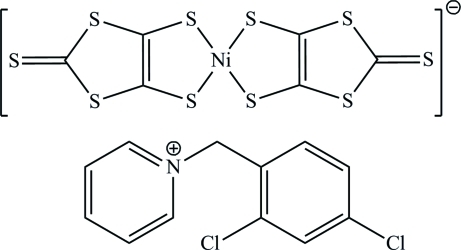


## Experimental

### 

#### Crystal data

(C_12_H_10_Cl_2_N)[Ni(C_3_S_5_)_2_]
*M* *_r_* = 690.48
Monoclinic, 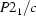

*a* = 14.4614 (5) Å
*b* = 8.2158 (3) Å
*c* = 21.8894 (8) Å
β = 107.231 (1)°
*V* = 2484.00 (15) Å^3^
*Z* = 4
Mo *K*α radiation
μ = 1.85 mm^−1^
*T* = 293 K
0.26 × 0.12 × 0.10 mm

#### Data collection

Bruker SMART APEX CCD area-detector diffractometer
Absorption correction: multi-scan (*SADABS*; Bruker, 2000 ▶) *T*_min_ = 0.645, *T*_max_ = 0.837
18903 measured reflections
4615 independent reflections
3992 reflections with *I* > 2σ(*I*)
*R*_int_ = 0.085

#### Refinement

*R*[*F* ^2^ > 2σ(*F* ^2^)] = 0.034
*wR*(*F* ^2^) = 0.092
*S* = 1.04
4615 reflections
290 parameters
H-atom parameters constrained
Δρ_max_ = 0.59 e Å^−3^
Δρ_min_ = −0.69 e Å^−3^

### 

Data collection: *SMART* (Bruker, 2000 ▶); cell refinement: *SAINT* (Bruker, 2000 ▶); data reduction: *SAINT*; program(s) used to solve structure: *SHELXS97* (Sheldrick, 2008 ▶); program(s) used to refine structure: *SHELXL97* (Sheldrick, 2008 ▶); molecular graphics: *SHELXTL* (Sheldrick, 2008 ▶); software used to prepare material for publication: *SHELXTL*.

## Supplementary Material

Crystal structure: contains datablock(s) I, global. DOI: 10.1107/S1600536811045004/gk2414sup1.cif

Structure factors: contains datablock(s) I. DOI: 10.1107/S1600536811045004/gk2414Isup2.hkl

Additional supplementary materials:  crystallographic information; 3D view; checkCIF report

## Figures and Tables

**Fig. 1.**
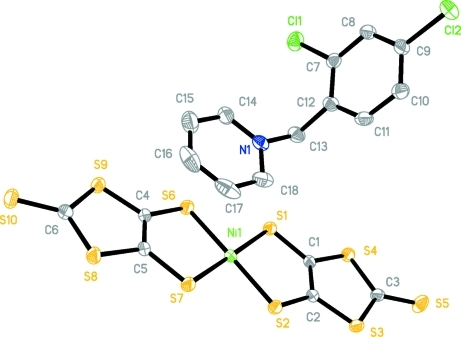
The cation and anion in the title compound with displacement ellipsoids drawn at the30% probability level. Hydrogen atoms have been omitted for clarity.

**Fig. 2.**
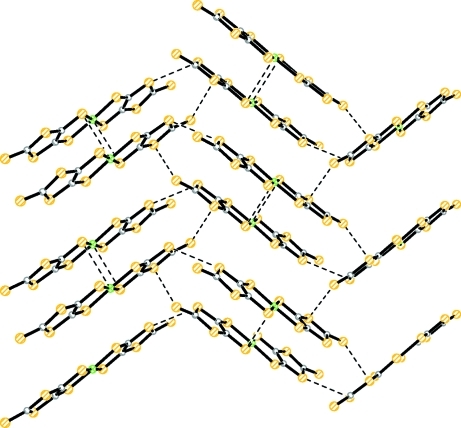
Two-dimensional supramolecular structure of [Ni(dmit)2]- anions through S···S and Ni···S contacts. Dashed lines indicate weak interactions.

## supplementary crystallographic information

### Comment

The bis(dithiolate)-metal complexes and their analogues with interesting structures and/or potential applications such as conducting/magnetic or non-linear optical (NLO) materials have been reported in recent years (Cassoux, 1999). We report herein the crystal structure of the title bis-dithiolate-metal complex. In this compound, the Ni^II^ cations of NiCl_2_.6H_2_O have been oxidized to Ni^III ^cation by I_3_^-^ (Cassoux *et al.*, 1991). The Ni^III ^cation is coordinated with two dmit^2-^ anions. As shown in Fig. 1, the asymmetric unit of the title compound contains one [Ni(dmit)_2_]^-^ anion and one [DiClPy]+ cation. Each nickel(III) ion is coordinated by four S atoms from two dmit ligands to complete a square-planar geometry, with Ni—S bond lengths ranging from 2.1589 (7) to 2.1640 (7) Å. The [Ni(dmit)_2_]^-^ anions related by inversion center form dimers with the S atom of one anion placed directly above the Ni atom of  another anion with Ni···S distance of 3.566 (2)Å (Ni1···S2^i: ^symmetry code: (i) -*x*, -*y*, -*z*], indicating the existence of electrostatic  Ni···S interactions.  The dimers linked through S3···S5^ii^ [3.6212 (11)Å] and S4···S8^iii^ [3.5573 (9)Å] [symmetry code: (ii) -*x*, 1/2 + *y*, 1/2 - *z*; (iii) *x*, 1/2 - *y*;1/2 + *z*] interactions form a two-dimensional layer structure, as depicted in Fig 2. The (C_12_H_10_Cl_2_N)^+^ cation adopts a Λ-shaped conformation, and the dihedral angles formed by the C12/C13/N1 plane with the benzene and pyridinium rings are 76.44 (2) and 86.75 (2)°, respectively.

### Experimental

4,5-Di(thiobenzoyl)-1,3-dithiole-2-thione (812 mg, 2 mmol) was suspended in methanol (10 ml). Sodium methoxide in methanol (prepared form 184 mg of sodium in 10 ml of methanol) was added to the above mixture under argon atmosphere at room temperature from 30 min to give a dark red solution. To this solution, NiCl_2_.6H_2_O (238 mg, 1 mmol) was added. After 30 min, a solution of I_2_ (127 mg, 1 mmol) and NaI (150 mg, 1 mmol) in methanol (20 ml) was added (the monoanionic [Ni(dmit)_2_]^-^ are obtained from the dianionic [Ni(dmit)_2_]^2-^ by I_3_^-^ oxidation). After another 10 min, a solution of 1-(2,4-dichlorobenzyl)pyridinium bromide [(DiClPy)Br] (317 mg, 1 mmol) in methanol (20 ml) was added to the reaction mixture. The solution was stirred for 30 min and cooled in a refrigerator overnight. The resultant dark green crystal was collected by filtration, and purified by recrystallization using a mixed solvent of acetonitrile and benzene.

### Refinement

H atoms were positioned geometrically, with C—H = 0.93 and 0.97 Å for aromatic and methylene H atoms, respectively, and constrained to ride on their parent atoms, with *U*_iso_(H) = 1.5Ueq(C).

### Crystal data

**Table:**

(C_12_H_10_Cl_2_N)[Ni(C_3_S_5_)_2_]	*F*(000) = 1388
*M**_r_* = 690.48	*D*_x_ = 1.846 Mg m^−^^3^
Monoclinic, *P*2_1_/*c*	Mo *K*α radiation, λ = 0.71073 Å
Hall symbol: -P 2ybc	Cell parameters from 9920 reflections
*a* = 14.4614 (5) Å	θ = 2.7–27.6°
*b* = 8.2158 (3) Å	µ = 1.85 mm^−^^1^
*c* = 21.8894 (8) Å	*T* = 293 K
β = 107.231 (1)°	Needle, black
*V* = 2484.00 (15) Å^3^	0.26 × 0.12 × 0.10 mm
*Z* = 4	

### Data collection

**Table:**

Bruker SMART APEX CCD area-detector diffractometer	4615 independent reflections
Radiation source: sealed tube	3992 reflections with *I* > 2σ(*I*)
graphite	*R*_int_ = 0.085
phi and ω scans	θ_max_ = 25.5°, θ_min_ = 2.0°
Absorption correction: multi-scan (*SADABS*; Bruker, 2000)	*h* = −14→17
*T*_min_ = 0.645, *T*_max_ = 0.837	*k* = −9→9
18903 measured reflections	*l* = −26→26

### Refinement

**Table:**

Refinement on *F*^2^	Secondary atom site location: difference Fourier map
Least-squares matrix: full	Hydrogen site location: inferred from neighbouring sites
*R*[*F*^2^ > 2σ(*F*^2^)] = 0.034	H-atom parameters constrained
*wR*(*F*^2^) = 0.092	*w* = 1/[σ^2^(*F*_o_^2^) + (0.0486*P*)^2^ + 0.8363*P*] where *P* = (*F*_o_^2^ + 2*F*_c_^2^)/3
*S* = 1.04	(Δ/σ)_max_ < 0.001
4615 reflections	Δρ_max_ = 0.59 e Å^−^^3^
290 parameters	Δρ_min_ = −0.69 e Å^−^^3^
0 restraints	Extinction correction: *SHELXL97* (Sheldrick, 2008), Fc^*^=kFc[1+0.001xFc^2^λ^3^/sin(2θ)]^-1/4^
Primary atom site location: structure-invariant direct methods	Extinction coefficient: 0.0070 (5)

### Special details

**Table:**

Geometry. All e.s.d.'s (except the e.s.d. in the dihedral angle between two l.s. planes) are estimated using the full covariance matrix. The cell e.s.d.'s are taken into account individually in the estimation of e.s.d.'s in distances, angles and torsion angles; correlations between e.s.d.'s in cell parameters are only used when they are defined by crystal symmetry. An approximate (isotropic) treatment of cell e.s.d.'s is used for estimating e.s.d.'s involving l.s. planes.
Refinement. Refinement of *F*^2^ against ALL reflections. The weighted *R*-factor *wR* and goodness of fit *S* are based on *F*^2^, conventional *R*-factors *R* are based on *F*, with *F* set to zero for negative *F*^2^. The threshold expression of *F*^2^ > σ(*F*^2^) is used only for calculating *R*-factors(gt) *etc*. and is not relevant to the choice of reflections for refinement. *R*-factors based on *F*^2^ are statistically about twice as large as those based on *F*, and *R*- factors based on ALL data will be even larger.

### Fractional atomic coordinates and isotropic or equivalent isotropic displacement parameters (Å^2^)

**Table:**

	*x*	*y*	*z*	*U*_iso_*/*U*_eq_	
Ni1	0.11672 (2)	0.16527 (4)	0.035816 (14)	0.03166 (12)	
S1	0.17227 (5)	−0.00117 (9)	0.11477 (3)	0.03781 (17)	
S2	−0.02108 (5)	0.18995 (9)	0.05446 (3)	0.04024 (18)	
S3	−0.10016 (4)	0.02836 (9)	0.15211 (3)	0.03853 (18)	
S4	0.07706 (5)	−0.14743 (9)	0.20813 (3)	0.03956 (18)	
S5	−0.07945 (6)	−0.17259 (11)	0.26887 (4)	0.0588 (2)	
S6	0.25347 (5)	0.13324 (9)	0.01601 (3)	0.04044 (18)	
S7	0.06384 (5)	0.33985 (9)	−0.04061 (3)	0.03918 (18)	
S8	0.15951 (5)	0.48291 (9)	−0.13470 (3)	0.04262 (19)	
S9	0.33185 (5)	0.28997 (9)	−0.08385 (3)	0.04294 (19)	
S10	0.31807 (6)	0.49623 (10)	−0.19673 (4)	0.0494 (2)	
C1	0.07448 (18)	−0.0249 (3)	0.14306 (11)	0.0311 (5)	
C2	−0.00959 (17)	0.0580 (3)	0.11635 (11)	0.0319 (5)	
C3	−0.03719 (18)	−0.1004 (3)	0.21241 (12)	0.0372 (6)	
C4	0.24237 (18)	0.2678 (3)	−0.04584 (11)	0.0337 (5)	
C5	0.16105 (18)	0.3563 (3)	−0.07046 (11)	0.0326 (5)	
C6	0.27173 (19)	0.4270 (3)	−0.14143 (12)	0.0375 (6)	
C7	0.47163 (18)	0.4200 (3)	0.30763 (12)	0.0369 (6)	
C8	0.5031 (2)	0.5037 (3)	0.36482 (13)	0.0424 (7)	
H8	0.5673	0.4975	0.3900	0.051*	
C9	0.4369 (2)	0.5968 (4)	0.38364 (13)	0.0427 (6)	
C10	0.3420 (2)	0.6087 (4)	0.34687 (14)	0.0459 (7)	
H10	0.2983	0.6730	0.3599	0.055*	
C11	0.3131 (2)	0.5234 (4)	0.29039 (14)	0.0452 (7)	
H11	0.2489	0.5314	0.2653	0.054*	
C12	0.37567 (19)	0.4262 (3)	0.26942 (12)	0.0378 (6)	
C13	0.3383 (2)	0.3300 (3)	0.20858 (13)	0.0446 (7)	
H13A	0.3840	0.2442	0.2077	0.054*	
H13B	0.2773	0.2793	0.2076	0.054*	
C14	0.3970 (2)	0.4626 (4)	0.12692 (14)	0.0505 (7)	
H14	0.4570	0.4154	0.1461	0.061*	
C15	0.3849 (3)	0.5594 (5)	0.07450 (15)	0.0648 (9)	
H15	0.4362	0.5777	0.0579	0.078*	
C16	0.2968 (4)	0.6292 (5)	0.04666 (16)	0.0733 (11)	
H16	0.2878	0.6969	0.0113	0.088*	
C17	0.2219 (3)	0.5989 (4)	0.07112 (16)	0.0687 (11)	
H17	0.1614	0.6451	0.0522	0.082*	
C18	0.2361 (2)	0.5005 (4)	0.12343 (15)	0.0533 (8)	
H18	0.1851	0.4790	0.1400	0.064*	
Cl1	0.55699 (6)	0.30754 (10)	0.28410 (4)	0.0559 (2)	
Cl2	0.47396 (7)	0.70086 (12)	0.45557 (4)	0.0695 (3)	
N1	0.32373 (16)	0.4347 (3)	0.15097 (10)	0.0396 (5)	

### Atomic displacement parameters (Å^2^)

**Table:**

	*U*^11^	*U*^22^	*U*^33^	*U*^12^	*U*^13^	*U*^23^
Ni1	0.02757 (19)	0.0410 (2)	0.02521 (18)	0.00030 (13)	0.00600 (13)	0.00174 (13)
S1	0.0295 (3)	0.0497 (4)	0.0341 (3)	0.0068 (3)	0.0091 (3)	0.0074 (3)
S2	0.0306 (3)	0.0546 (4)	0.0353 (3)	0.0089 (3)	0.0092 (3)	0.0151 (3)
S3	0.0270 (3)	0.0512 (4)	0.0370 (4)	0.0023 (3)	0.0087 (3)	0.0107 (3)
S4	0.0352 (4)	0.0480 (4)	0.0353 (3)	0.0090 (3)	0.0100 (3)	0.0125 (3)
S5	0.0494 (5)	0.0755 (6)	0.0598 (5)	0.0147 (4)	0.0289 (4)	0.0336 (4)
S6	0.0314 (3)	0.0527 (4)	0.0376 (4)	0.0076 (3)	0.0108 (3)	0.0118 (3)
S7	0.0300 (3)	0.0521 (4)	0.0365 (3)	0.0072 (3)	0.0114 (3)	0.0102 (3)
S8	0.0403 (4)	0.0524 (4)	0.0373 (4)	0.0070 (3)	0.0149 (3)	0.0117 (3)
S9	0.0341 (4)	0.0571 (5)	0.0409 (4)	0.0063 (3)	0.0161 (3)	0.0082 (3)
S10	0.0524 (5)	0.0580 (5)	0.0453 (4)	−0.0010 (3)	0.0263 (4)	0.0070 (3)
C1	0.0312 (13)	0.0358 (13)	0.0250 (11)	0.0017 (10)	0.0060 (10)	0.0011 (10)
C2	0.0317 (13)	0.0371 (14)	0.0259 (11)	−0.0016 (10)	0.0072 (10)	−0.0007 (10)
C3	0.0309 (13)	0.0436 (15)	0.0351 (13)	−0.0004 (11)	0.0067 (11)	0.0040 (12)
C4	0.0310 (13)	0.0433 (14)	0.0272 (12)	−0.0017 (11)	0.0094 (10)	−0.0028 (11)
C5	0.0334 (13)	0.0395 (14)	0.0255 (12)	−0.0016 (11)	0.0097 (10)	0.0009 (10)
C6	0.0367 (14)	0.0404 (15)	0.0346 (13)	−0.0036 (11)	0.0093 (11)	−0.0030 (11)
C7	0.0343 (14)	0.0400 (15)	0.0369 (13)	−0.0001 (11)	0.0116 (11)	0.0064 (11)
C8	0.0355 (15)	0.0531 (18)	0.0359 (14)	−0.0070 (12)	0.0065 (12)	0.0017 (12)
C9	0.0455 (16)	0.0477 (16)	0.0350 (14)	−0.0146 (13)	0.0120 (12)	−0.0065 (12)
C10	0.0398 (16)	0.0488 (17)	0.0505 (17)	−0.0050 (13)	0.0156 (13)	−0.0090 (14)
C11	0.0291 (14)	0.0540 (18)	0.0481 (16)	−0.0027 (12)	0.0045 (12)	−0.0045 (13)
C12	0.0397 (15)	0.0354 (14)	0.0362 (14)	−0.0080 (11)	0.0080 (11)	0.0014 (11)
C13	0.0484 (17)	0.0397 (15)	0.0416 (15)	−0.0063 (12)	0.0070 (13)	−0.0025 (12)
C14	0.0462 (17)	0.067 (2)	0.0377 (15)	0.0049 (15)	0.0109 (13)	−0.0032 (14)
C15	0.078 (3)	0.076 (2)	0.0428 (17)	−0.004 (2)	0.0214 (17)	0.0046 (17)
C16	0.115 (3)	0.057 (2)	0.0365 (17)	0.000 (2)	0.006 (2)	0.0055 (15)
C17	0.070 (2)	0.059 (2)	0.053 (2)	0.0159 (18)	−0.0184 (18)	−0.0065 (17)
C18	0.0400 (17)	0.0571 (19)	0.0533 (18)	0.0019 (14)	−0.0008 (14)	−0.0100 (15)
Cl1	0.0503 (4)	0.0688 (5)	0.0517 (4)	0.0169 (4)	0.0200 (4)	0.0074 (4)
Cl2	0.0677 (6)	0.0911 (7)	0.0480 (5)	−0.0198 (5)	0.0144 (4)	−0.0287 (4)
N1	0.0406 (13)	0.0401 (13)	0.0336 (11)	−0.0008 (10)	0.0037 (10)	−0.0076 (10)

### Geometric parameters (Å, °)

**Table:**

Ni1—S2	2.1589 (7)	C8—C9	1.381 (4)
Ni1—S6	2.1624 (7)	C8—H8	0.9300
Ni1—S1	2.1633 (7)	C9—C10	1.374 (4)
Ni1—S7	2.1640 (7)	C9—Cl2	1.732 (3)
S1—C1	1.715 (2)	C10—C11	1.374 (4)
S2—C2	1.704 (2)	C10—H10	0.9300
S3—C3	1.726 (3)	C11—C12	1.384 (4)
S3—C2	1.731 (2)	C11—H11	0.9300
S4—C3	1.726 (3)	C12—C13	1.505 (4)
S4—C1	1.736 (2)	C13—N1	1.489 (3)
S5—C3	1.643 (3)	C13—H13A	0.9700
S6—C4	1.718 (3)	C13—H13B	0.9700
S7—C5	1.722 (2)	C14—N1	1.335 (4)
S8—C6	1.734 (3)	C14—C15	1.364 (4)
S8—C5	1.744 (2)	C14—H14	0.9300
S9—C6	1.721 (3)	C15—C16	1.365 (5)
S9—C4	1.743 (2)	C15—H15	0.9300
S10—C6	1.649 (3)	C16—C17	1.365 (6)
C1—C2	1.365 (3)	C16—H16	0.9300
C4—C5	1.352 (4)	C17—C18	1.367 (5)
C7—C8	1.382 (4)	C17—H17	0.9300
C7—C12	1.393 (4)	C18—N1	1.345 (4)
C7—Cl1	1.738 (3)	C18—H18	0.9300
			
S2—Ni1—S6	178.26 (3)	C7—C8—H8	120.8
S2—Ni1—S1	93.06 (3)	C10—C9—C8	121.7 (3)
S6—Ni1—S1	86.45 (3)	C10—C9—Cl2	118.9 (2)
S2—Ni1—S7	87.10 (3)	C8—C9—Cl2	119.3 (2)
S6—Ni1—S7	93.46 (3)	C9—C10—C11	118.4 (3)
S1—Ni1—S7	177.67 (3)	C9—C10—H10	120.8
C1—S1—Ni1	102.09 (8)	C11—C10—H10	120.8
C2—S2—Ni1	102.27 (9)	C10—C11—C12	122.6 (3)
C3—S3—C2	97.68 (12)	C10—C11—H11	118.7
C3—S4—C1	97.25 (12)	C12—C11—H11	118.7
C4—S6—Ni1	101.67 (9)	C11—C12—C7	117.1 (2)
C5—S7—Ni1	101.78 (9)	C11—C12—C13	119.9 (2)
C6—S8—C5	96.99 (12)	C7—C12—C13	123.0 (3)
C6—S9—C4	97.61 (12)	N1—C13—C12	111.7 (2)
C2—C1—S1	120.93 (19)	N1—C13—H13A	109.3
C2—C1—S4	116.16 (19)	C12—C13—H13A	109.3
S1—C1—S4	122.87 (14)	N1—C13—H13B	109.3
C1—C2—S2	121.51 (19)	C12—C13—H13B	109.3
C1—C2—S3	115.69 (19)	H13A—C13—H13B	107.9
S2—C2—S3	122.71 (15)	N1—C14—C15	120.8 (3)
S5—C3—S3	124.54 (16)	N1—C14—H14	119.6
S5—C3—S4	122.34 (16)	C15—C14—H14	119.6
S3—C3—S4	113.12 (14)	C14—C15—C16	119.4 (3)
C5—C4—S6	121.83 (19)	C14—C15—H15	120.3
C5—C4—S9	115.77 (19)	C16—C15—H15	120.3
S6—C4—S9	122.29 (15)	C15—C16—C17	119.5 (3)
C4—C5—S7	121.21 (19)	C15—C16—H16	120.3
C4—C5—S8	116.37 (19)	C17—C16—H16	120.3
S7—C5—S8	122.40 (15)	C16—C17—C18	119.8 (3)
S10—C6—S9	122.42 (16)	C16—C17—H17	120.1
S10—C6—S8	124.40 (16)	C18—C17—H17	120.1
S9—C6—S8	113.18 (15)	N1—C18—C17	120.0 (3)
C8—C7—C12	121.9 (3)	N1—C18—H18	120.0
C8—C7—Cl1	117.4 (2)	C17—C18—H18	120.0
C12—C7—Cl1	120.7 (2)	C14—N1—C18	120.5 (3)
C9—C8—C7	118.3 (3)	C14—N1—C13	120.2 (2)
C9—C8—H8	120.8	C18—N1—C13	119.3 (3)
			
C12—C7—C8—C9	−0.5 (4)	C11—C12—C13—N1	77.2 (3)
Cl1—C7—C8—C9	178.5 (2)	C7—C12—C13—N1	−104.5 (3)
C7—C8—C9—C10	−0.6 (4)	N1—C14—C15—C16	0.4 (5)
C7—C8—C9—Cl2	179.2 (2)	C14—C15—C16—C17	−1.0 (5)
C8—C9—C10—C11	0.8 (4)	C15—C16—C17—C18	0.6 (5)
Cl2—C9—C10—C11	−179.0 (2)	C16—C17—C18—N1	0.5 (5)
C9—C10—C11—C12	0.2 (4)	C15—C14—N1—C18	0.7 (4)
C10—C11—C12—C7	−1.3 (4)	C15—C14—N1—C13	−179.4 (3)
C10—C11—C12—C13	177.2 (3)	C17—C18—N1—C14	−1.1 (4)
C8—C7—C12—C11	1.5 (4)	C17—C18—N1—C13	179.0 (3)
Cl1—C7—C12—C11	−177.6 (2)	C12—C13—N1—C14	86.7 (3)
C8—C7—C12—C13	−176.9 (3)	C12—C13—N1—C18	−93.4 (3)
Cl1—C7—C12—C13	4.0 (4)		
